# Supplementary material for: Crystal structure of the plant symporter STP10 illuminates sugar uptake mechanism in monosaccharide transporter superfamily
Source: Nat Commun. 2019 Jan 24;10:407. doi: 10.1038/s41467-018-08176-9 (PMC6345825; doi:10.1038/s41467-018-08176-9)
Supplement: Supplementary file 2 — Supplementary Information [file 41467_2018_8176_MOESM2_ESM.pdf]

SUPPLEMENTARY MATERIAL

Crystal structure of the plant symporter STP10 illuminates sugar uptake mechanism in Monosaccharide Transporter Superfamily

Peter Aasted Paulsen[1], Tânia F. Custódio[1], Bjørn Panyella Pedersen[1,2]

1) Department of Molecular Biology and Genetics, Aarhus University, Gustav Wieds Vej 10, DK-8000 Aarhus C, Denmark.

2) Aarhus Institute of Advanced Studies, Aarhus University, Høegh-Guldbergs Gade 6B, DK-8000 Aarhus C, Denmark.

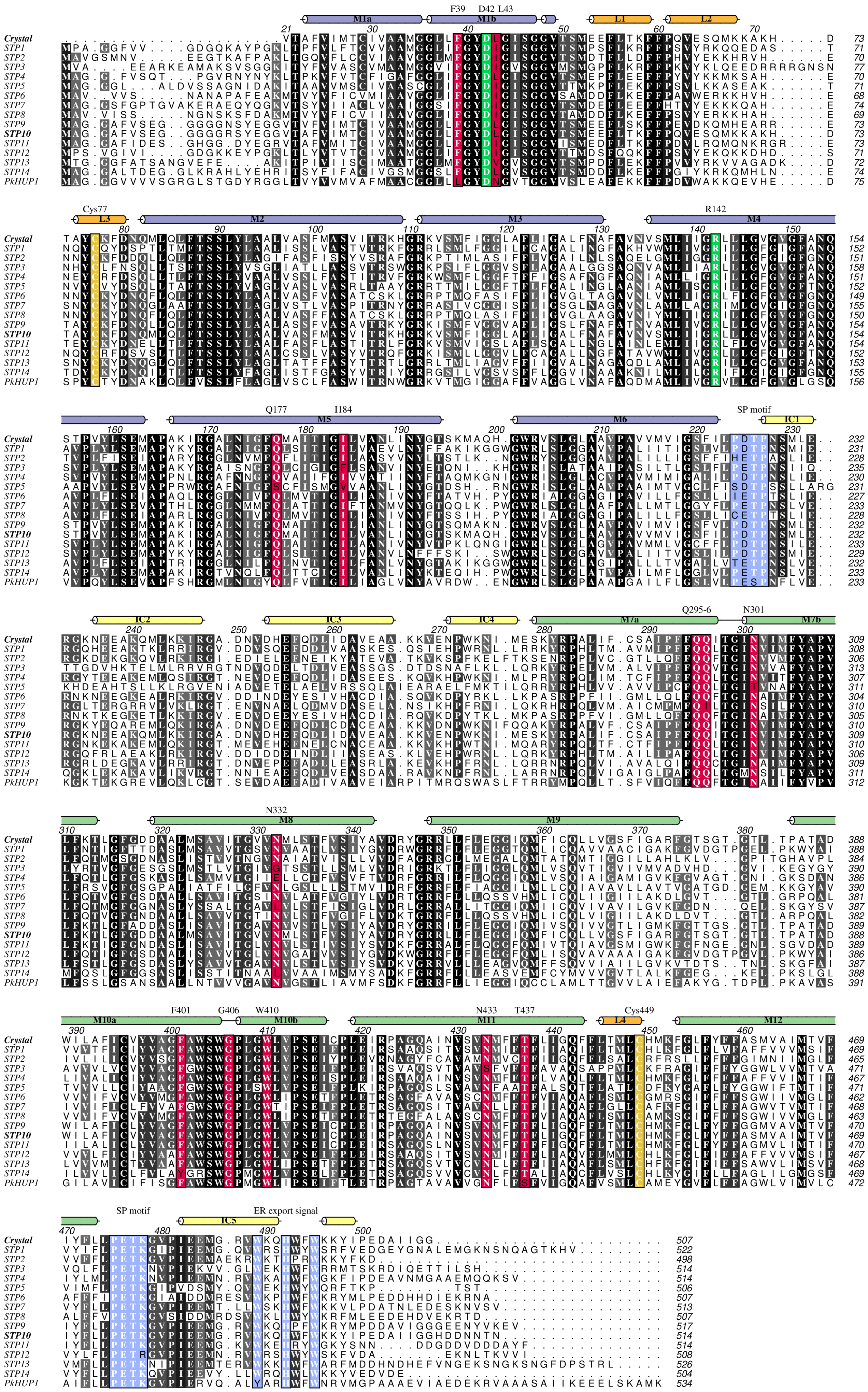

F39
D42 L43
M1a
M1b
L1
L2
Cys77
L3
M2
M3
R142
M4
Q177
I184
M5
M6
SP motif
IC1
IC2
IC3
IC4
M7a
Q295-6
N301
M7b
N332
M8
M9
M10a
F401
G406
W410
M10b
N433
T437
M11
Cys449
L4
M12
SP motif
IC5
ER export signal
Crystal
STP1
STP2
STP3
STP4
STP5
STP6
STP7
STP8
STP9
STP10
STP11
STP12
STP13
STP14
PkHUP1

Supplementary Figure 1. Multiple sequence alignment of the *A. thaliana* Sugar Transport Family, with *Parachlorella kessleri* HUP1 included. Alignment between STP1 (accession number P23586), STP2 (accession number Q9LNV3), STP3 (accession number Q8L7R8), STP4 (accession number Q39228), STP5 (accession number Q93Y91), STP6 (accession number Q9SFG0), STP7 (accession number O04249), STP8 (accession number Q9SBA7), STP9 (accession number Q9SX48), STP10 (accession number Q9LT15), STP11 (accession number Q9FMX3), STP12 (accession number O65413), STP13 (accession number Q94AZ2), STP14 (accession number Q8GW61), *Parachlorella kessleri* HUP1 (accession number P15686). Conserved residues are highlighted with gray-scale, where black is perfectly conserved. Colored tubes represent α-helices found in the N domain (blue), Lid domain (orange), ICH domain (pale yellow) and C domain (green). Key residues are numbered above the α-helix markings. Residues highlighted in dark red participate in sugar binding. The proton donor/acceptor pair is highlighted in green. The cysteines forming the disulfide bridge between Lid domain and C domain are highlighted in dark yellow. Conserved motifs are highlighted in light blue.

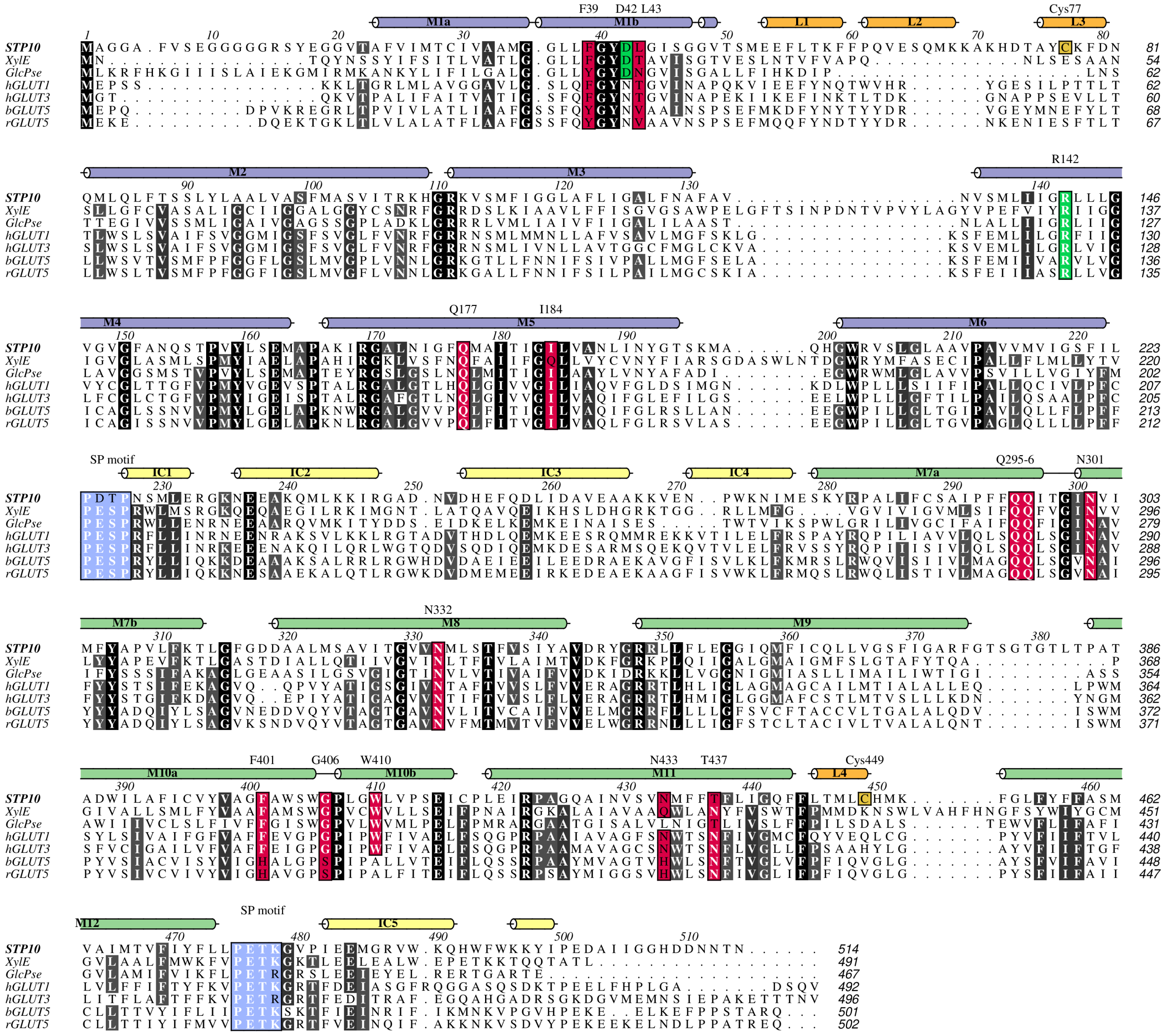

Supplementary Figure 2. Multiple sequence alignment of the *A. thaliana* STP10 with other Major Facilitator sugar transporters from the Sugar Porter group. Alignment between STP10 (accession number Q9LT15), XylE (accession number P0AGF4), GlcPse (accession number A0A0H2VG78), hGLUT1 (accession number P11166), hGLUT3 (accession number P11169), bGLUT5 (accession number P58353), rGLUT5 (accession number P43427). Conserved residues are highlighted with gray-scale, where black is perfectly conserved. Colored tubes represent α-helices found in the N domain (blue), Lid domain (orange), ICH domain (pale yellow) and C domain (green). Key residues are numbered above the α-helix markings. Residues highlighted in dark red participate in sugar binding. The proton donor/acceptor pair is highlighted in green. The cysteines forming the disulfide bridge between Lid domain and C domain are highlighted in dark yellow. Conserved motifs are highlighted in light blue.

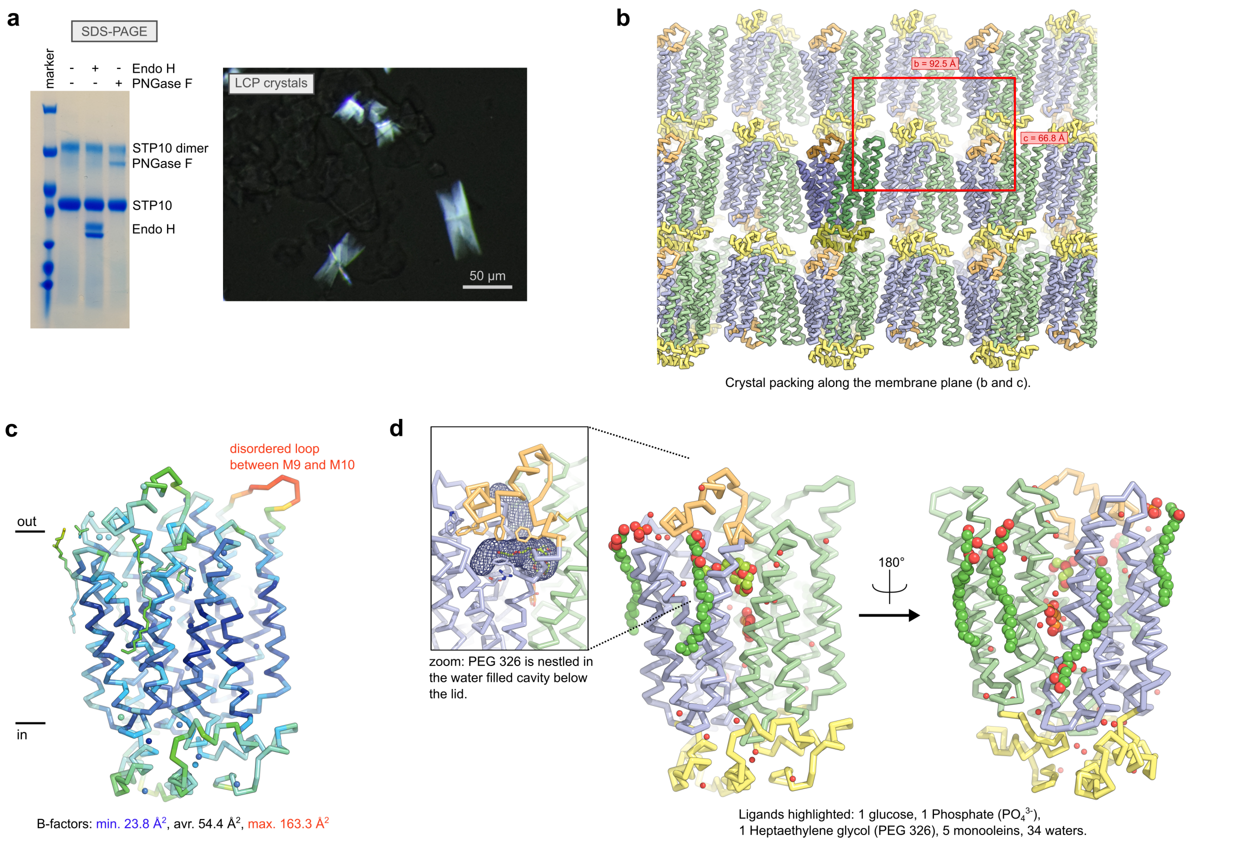

Supplementary Figure 3. Crystals and components of the asymmetric unit. a, SDS-PAGE gel of STP10 and polarized light photo of STP10 crystals. STP10 has a predicted glycosylation site at Asn134. While the crystal packing would allow the presence of glycosylation no density was observed, and incubating the STP10 sample over night with Endo H or PNGase F did not result in a mass-shift, indicating that STP10 is not glycosylated when overexpressed in yeast. **b**, Asymmetric unit and crystal packing of STP10. The unit cell is viewed perpendicular to the bc-plane, and the b and c axis highlighted in red. The asymmetric unit contains one molecule of STP10, as highlighted in darker colors. The packing is a nice example of type I packing normally obtained by LCP crystallography with the transmembrane regions packing in a lipid bilayer and a relatively low solvent content (54%). **c**, The backbone of STP10 colored by the atomic displacement factor (B-factor) with a rainbow gradient from low/blue (23.8 Å^2) to high/red (163.3 Å^2). There is a disordered loop between M9 and M10 with a significantly higher B-factor than the rest of the model. **d**, Backbone representation of STP10 with all the heterologous molecules found in the density highlighted. Besides STP10 we could confidently build 1 glucose, 1 phosphate, 1 PEG 326, 5 monoolein molecules and 34 waters.

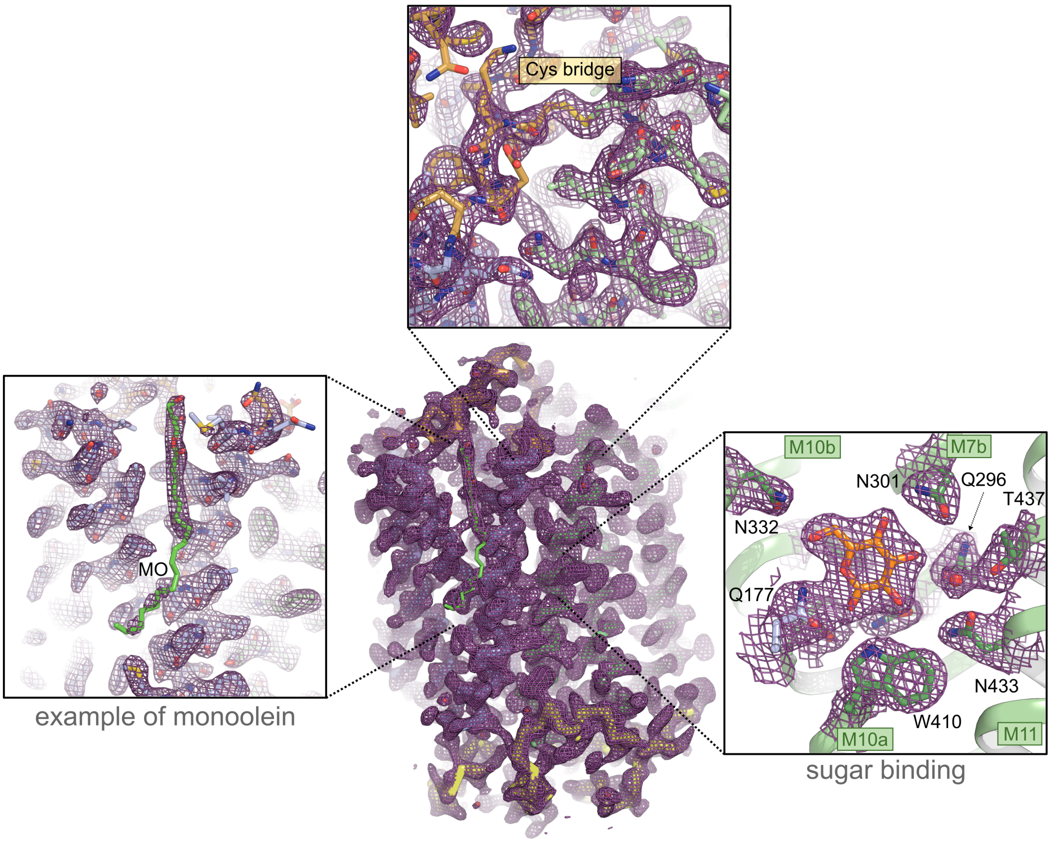

Supplementary Figure 4. Examples of electron density. 2mFo-DFc electron density calculated after the final refinement run (1.5 σ) in magenta with the final model overlaid. The three inserts highlight the high quality of the electron density for glucose binding and the disulfide bridge and an example of the density of the monooleine, which was weaker and is clearer at lower sigma levels than 1.5.

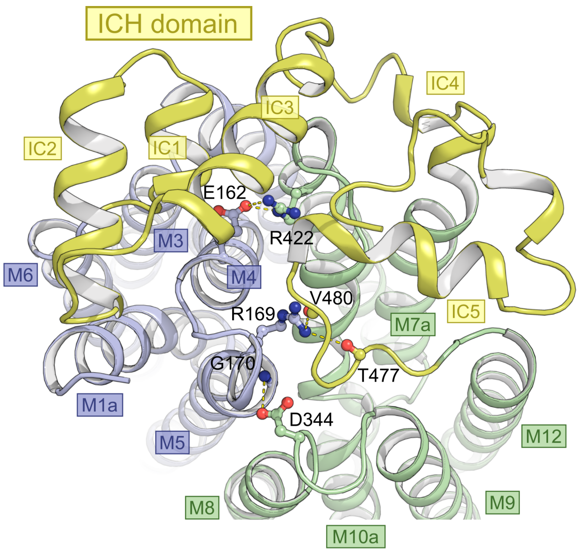

Supplementary Figure 5. Closure towards the cytosolic side in STP10. View of STP10 from the cytosolic side. The opening between the N and C domain towards the cytosolic is closed and held in place by several highlighted interactions between the two domains. In particular by a double salt bridge from D344(M8) to the main chain nitrogen of Gly170(M5), and from Arg169(M5) to the main chain carbonyls of Thr477 and Val480, as well as a salt bridge from Glu162 (M4) to Arg422(M11). These regions are perfectly conserved in all STPs and several bacterial symporters (Supplementary Figure 2), and have also been observed in human sugar facilitators.

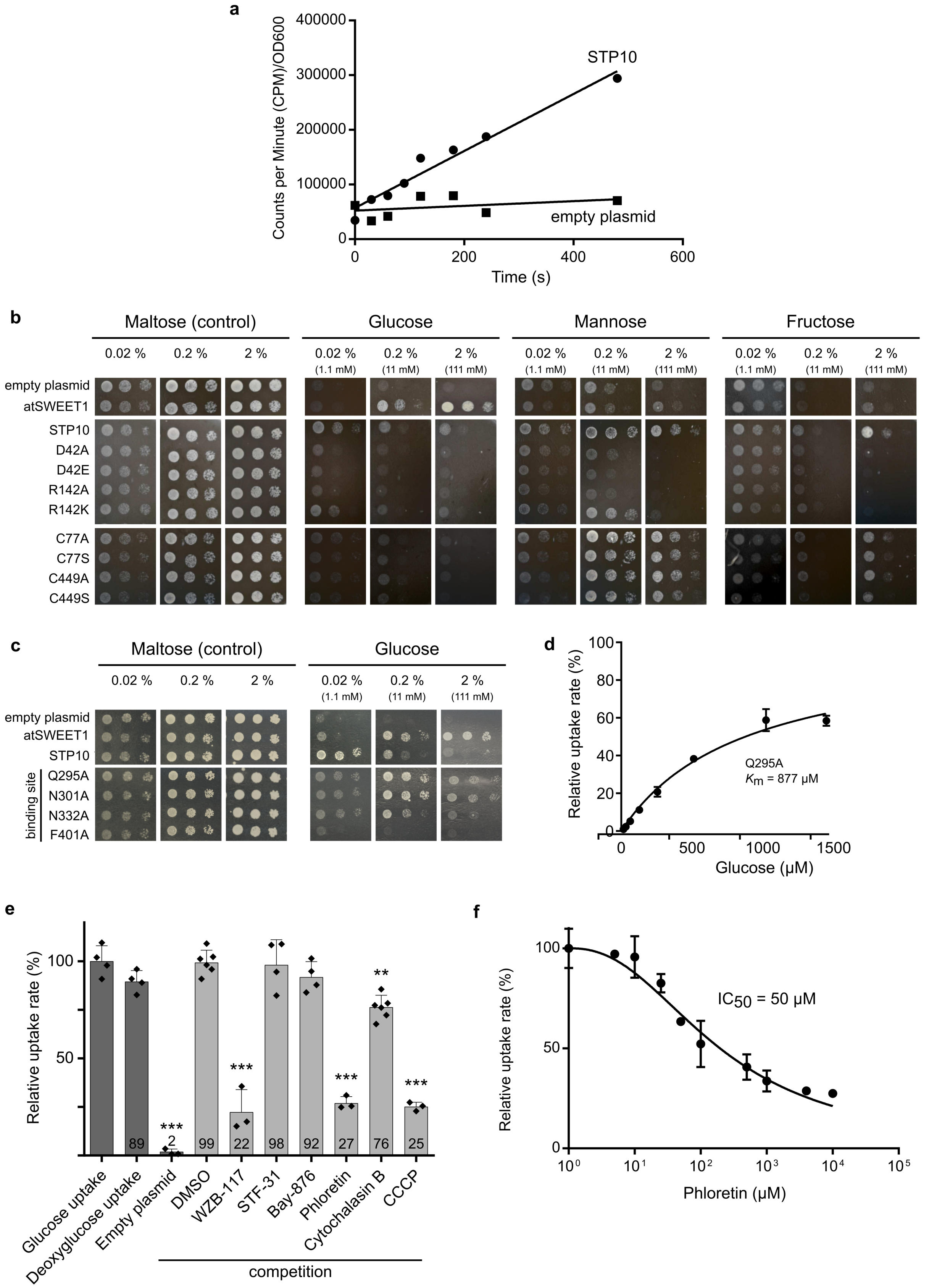

Supplementary Figure 6. Functional characterization of STP10. a, Uptake of glucose into EBY.VW4000 yeast strain expressing STP10 (circles) or empty plasmid (squares) per OD600 of cells at an initial outside concentration of 100 µM glucose at pH 5.0. **b**, Growth complementation of the *S. cerevisiae* hexose transport deficient strain, EBY.VW4000 by STP10, STP10 mutants and

Arabidopsis thaliana SWEET1 (positive control for glucose uptake) at different concentrations of sugars. **c**, Growth complementation of EBY.VW4000 by STP10 substrate binding site mutants. **d**, Determination of kinetic parameters for the transport of glucose measured using the EBY.VW4000 uptake assay for the mutant Q295A. Shown is a Michaelis-Menten fit to glucose titration. Data are mean ± SD of three or more replicate experiments. **e**, STP10 inhibition determined by the EBY.VW4000 competition assay at pH 5.0. *, $P \leq 0.05$; **, $P \leq 0.01$; and ***, $P \leq 0.001$ by Student's *t* test. **f**, Dose response curve showing inhibition of STP10 activity by Phloretin. Data for all assays are mean ± SD of three or more replicate experiments.

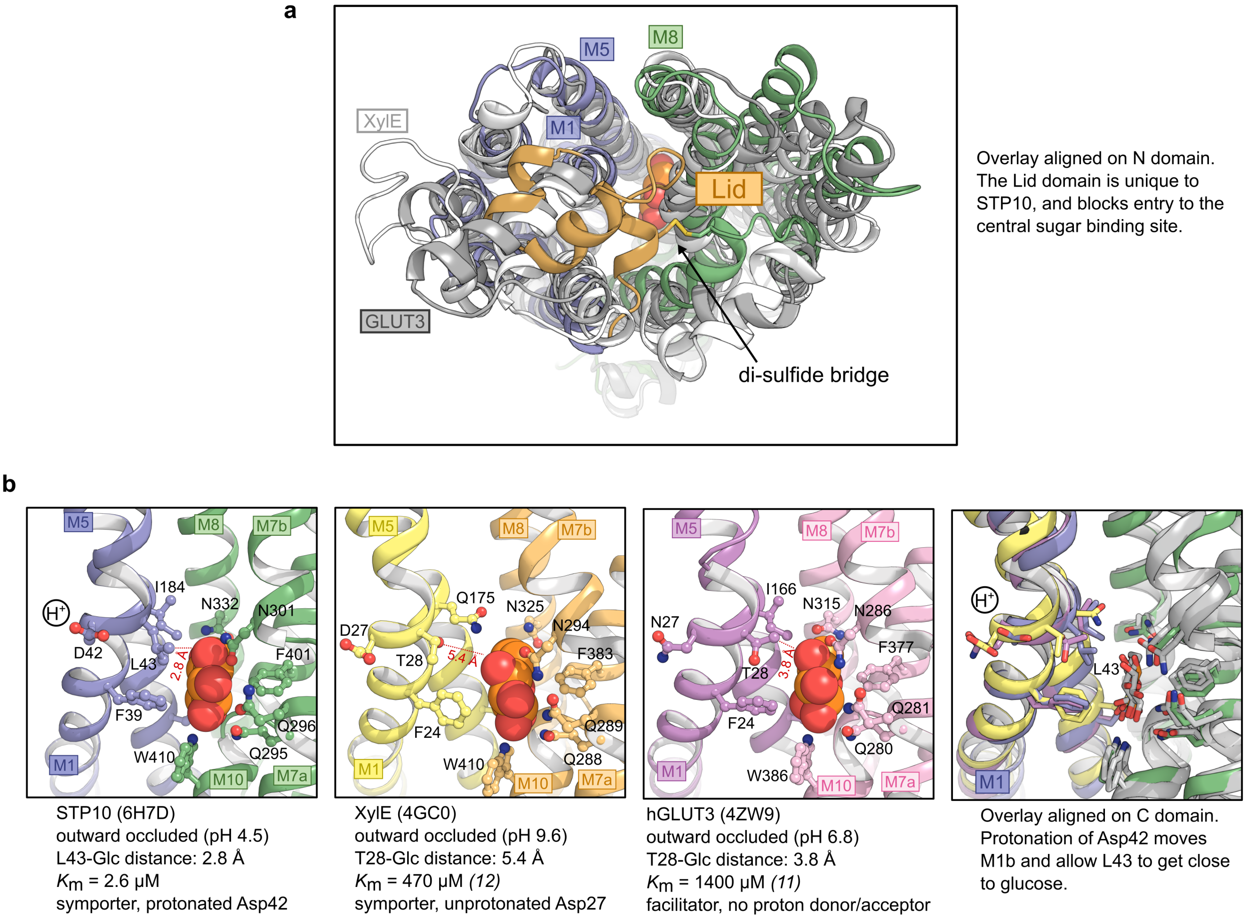

Supplementary Figure 7. Comparison of STP10 with XylE and hGLUT3. a, Overlay of the extracellular region in STP10, XylE and hGLUT3 (All in outward occluded conformations).
b, Comparison of the sugar binding sites in STP10, XylE and hGLUT3. All the polar interactions from the C domain are identical in the three cases, despite large differences in affinity. Differences are found in the N domain. In particular, the hydrophobic interaction between substrate and L43 in STP10 is replaced by a polar interaction from a Serine in XylE and Glut3. Only in STP10 is the proton donor/acceptor residue (Asp42) protonated, causing the M1 helix to bend away from Arg142 and move Leu43 towards the substrate.

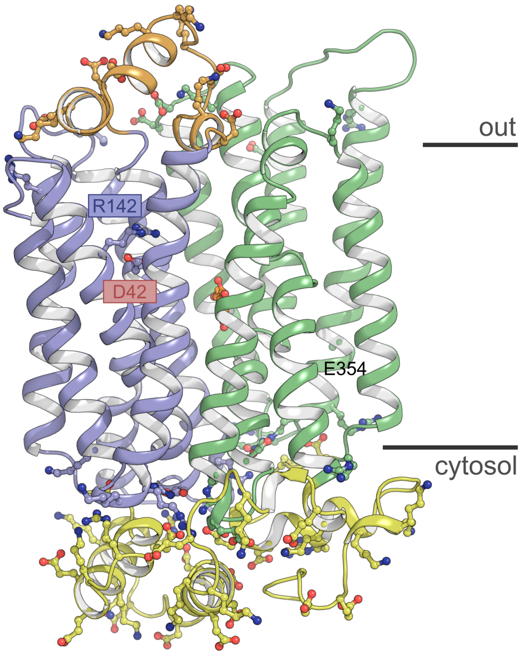

Supplementary Figure 8. Identification of all charged residues in STP10. STP10 is shown in cartoon with all charged residues shown as sticks. Asp/Glu are red while Lys/Arg are blue. Only Asp42 and Arg142 are buried in the membrane plane and in position to form the proton donor/acceptor pair. There is a Glu354 that is also in the membrane and pointing towards the solvent. It is highly unlikely that this residues (that is not conserved in STPs), would have a functional role, but it might be implicated in determining the energy landscape of conformational change.

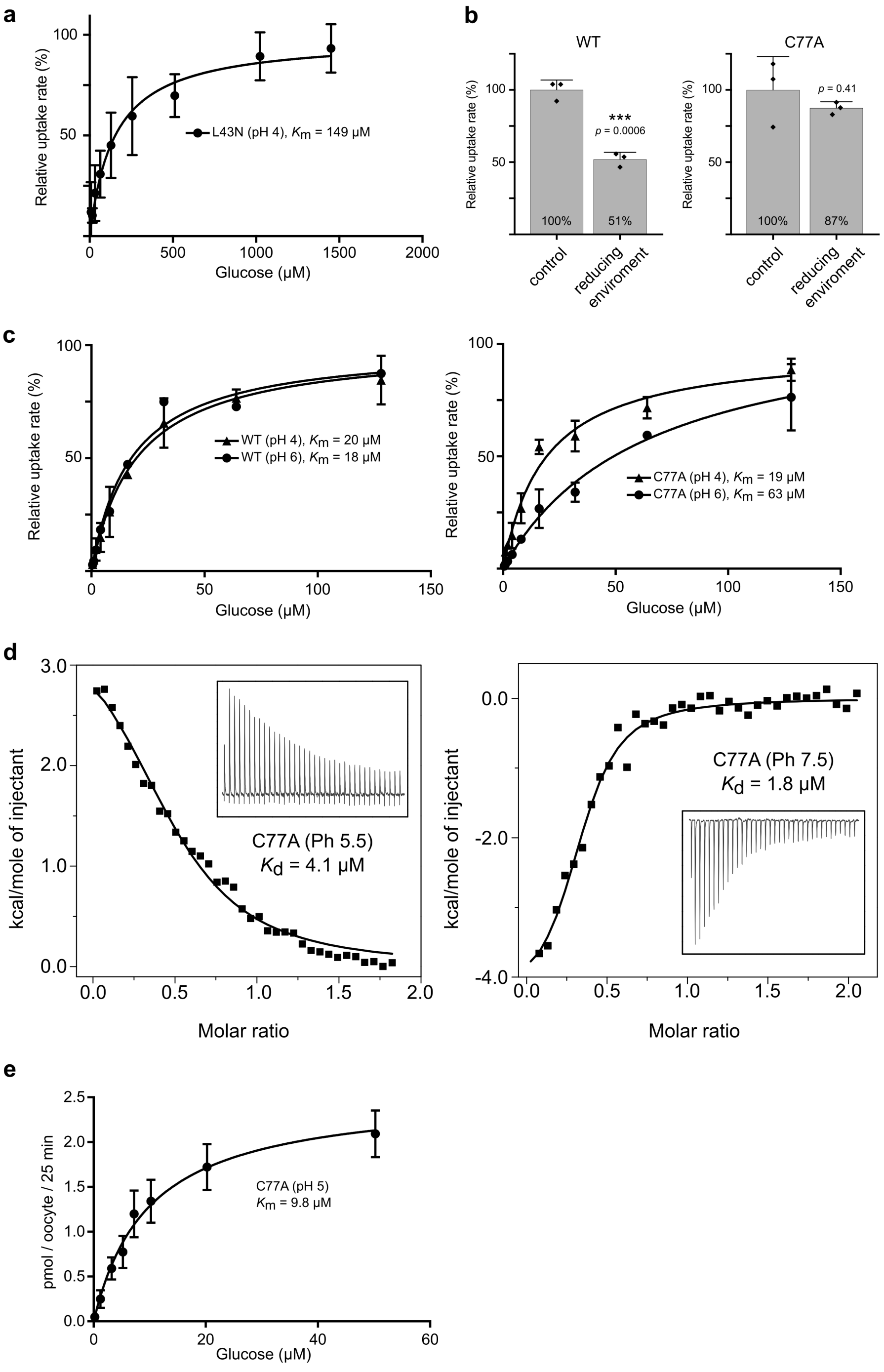

Supplementary Figure 9. Functional characterization of STP10 mutants L43N and C77A. a, Michaelis-Menten fit to glucose titration of STP10 mutant L43A at pH 4.0. **b**, Effect of reducing agent on glucose transport using the EBY.VW4000 uptake assay for WT and C77A. The uptake assay was done at pH 6.0 either under standard conditions or in a reducing environment (by addition of 2.5% (V/V) 2-Mercaptoethanol). ***, P <= 0.001 by Student's *t* test. **c**, Determination of kinetic parameters for the transport of glucose measured using the EBY.VW4000 uptake assay. Shown is a Michaelis-Menten fit to glucose titration at pH 4 and pH 6 (left) and for the mutant C77A at pH 4 and pH 6 (right). Data are mean ± SD of three or more replicate experiments. **d**, The

binding affinity between glucose and STP10-C77A was measured with Isothermal titration calorimetry at pH 5.5 and at pH 7.5. The apparent *K*d does not change with pH. **e**, Determination of kinetic parameters for the transport of glucose by STP10 C77A measured using the *Xenopus* oocyte uptake assay at pH 5. Shown is a Michaelis-Menten fit to glucose titration. Data for all assays are mean ± SD of three or more replicate experiments.

Supplementary Table 1. Sequence identity of STP10 to the *A. thaliana* Sugar Transport Family.

Name	Sequence %ID (Needle-Wunsch alignment)
STP1	59.4%
STP2	50.5%
STP3	46.5%
STP4	64.6%
STP5	48.2%
STP6	52.4%
STP7	53.7%
STP8	51.9%
STP9	84.3%
STP11	70.8%
STP12	60.5%
STP13	52.8%
STP14	50.7%

Supplementary Table 2. Sequence identity and Root Mean Square Deviation of STP10 to structures from the Sugar Porter group.

Name	PDB code	Sequence %ID (Needle-Wunsch alignment)	RMSD (A) (all-atom fit to STP10)
XylE	4GC0	27.1%	1.86
XylE	4JA3	27.1%	2.71
XylE	4JA4	27.1%	3.04
XylE	4QIQ	27.1%	2.93
GlcPse	4LDS	27.8%	3.31
hGLUT1	4PYP	23.4%	3.50
hGLUT3	4ZWC	25.2%	1.89
hGLUT3	4ZW9	25.2%	1.83
bGLUT5	4YB9	24.0%	3.34
rGLUT5	4YBQ	26.0%	2.12

Supplementary Table 3. Data collection and refinement statistics

	STP10:Glucose
**Data collection**	
Space group	P 2_1 (#4)
No of crystals merged	2
Cell dimensions	
a, *b*, *c* (Å)	51.7, 92.5, 66.8
α, β, γ (°)	90.0, 109.4, 90.0
Resolution (Å)	63 - 2.4 (2.5 - 2.4) *
R_{meas} (%)	30.6 (234.3)
$I / \sigma I$	4.9 (0.9)
CC(1/2) (%)	99.1 (34.8)
Completeness (%)	99.8 (99.8)
Redundancy	6.6 (6.6)
**Refinement**	
Resolution (Å)	63 - 2.4 (2.51 - 2.4)
No. reflections (work/free)	22,078 / 1,162
R_{work}	20.3 (31.7)
R_{free}	26.8 (36.6)
No. atoms	
Protein	3,771
Ligand/ion	164
Water	34
B-factors	
Protein	53.9
Ligand/ion	68.7
Water	46.4
R.m.s. deviations	
Bond lengths (Å)	0.01
Bond angles (°)	1.01
Ramachandran plot	
Favored (%)	95.7
Outliers (%)	0.0
Rotamer outliers (%)	0.25
Clashscore	10.38
Molprobity score	1.84

*Values in parentheses are for highest-resolution shell.
